# Muscular Sestrins: Roles in Exercise Physiology and Stress Resistance

**DOI:** 10.3390/biom13050722

**Published:** 2023-04-23

**Authors:** Irene Hwang, Myungjin Kim

**Affiliations:** Department of Molecular & Integrative Physiology, University of Michigan, Ann Arbor, MI 48109, USA

**Keywords:** muscle, Sestrin, exercise, physiology, stress, heart

## Abstract

Sestrins are a family of stress-inducible proteins that are critical for stress adaptation and the maintenance of metabolic homeostasis. High expression of Sestrins is observed in skeletal and cardiac muscle tissues, suggesting their significance in the physiological homeostasis of these organs. Furthermore, expression of Sestrins is dynamically controlled in the tissues, based on the level of physical activity and the presence or absence of stress insults. Genetic studies in model organisms have shown that muscular Sestrin expression is critical for metabolic homeostasis, exercise adaptation, stress resistance, and repair and may mediate the beneficial effects of some available therapeutics. The current minireview summarizes and discusses recent findings that shed light on the role of Sestrins in regulating muscle physiology and homeostasis.

## 1. Introduction: Sestrin, Its Structure and Biochemical Roles

Sestrins are a family of stress-inducible proteins that play a critical role in maintaining metabolic homeostasis and stress adaptation [1]. They were initially discovered as genes induced by the tumor suppressor p53 [2] and have since been found to be involved in various cellular processes, including autophagy, antioxidant defense, and mTORC1 signaling [3]. Most importantly, Sestrin expression is regulated through a diverse range of stress-inducible transcription factors, including p53, FoxO, Atf4, and Nrf2 [4]. Different stresses induce Sestrin expression through one or more of these transcription factors, which counteracts the stresses and their consequences, restoring homeostasis and completing the negative feedback loop. Sestrins can also be post-translationally controlled, such as through phosphorylation and ubiquitination [5].

Upon exposure to stress, Sestrins serve a variety of physiological roles in counteracting the damage inflicted by these stress insults. Initially, Sestrins were proposed as antioxidant molecules capable of recycling peroxiredoxins [6]. Although it was later demonstrated that physiological peroxiredoxin reduction is primarily mediated by a different enzyme [7], Sestrins still exhibit in vitro oxidoreductase activity towards reactive oxygen species [8], suggesting that they can indeed function as antioxidants.

In addition to their oxidoreductase activity, Sestrins possess numerous functions that are independent of this activity. For example, they have been shown to upregulate AMP-activated protein kinase (AMPK) and downregulate the mammalian target of rapamycin complex 1 (mTORC1) [3]. The exact mechanisms by which Sestrins upregulate AMPK remain unclear; however, once AMPK is upregulated, it phosphorylates and activates tuberous sclerosis complex 2 (TSC2), which subsequently inhibits mTORC1 activity [9]. Furthermore, an AMPK-independent mechanism has been identified in which Sestrins can inhibit GATOR2 complex activity, leading to the inhibition of GATOR1, which, in turn, suppresses mTORC1 [10,11,12]. As a result, Sestrins are capable of downregulating mTORC1 through two distinct mechanisms: the upregulation of AMPK and the inhibition of GATOR2.

Notably, the Sestrin-induced upregulation of AMPK and downregulation of mTORC1 can both contribute to the suppression of cellular anabolism and the activation of autophagic catabolism. While AMPK activates the autophagy-initiating protein kinase ULK1, mTORC1 inhibits it [13]. Consequently, it has been suggested that by promoting catabolism and suppressing anabolism, Sestrins help maintain cellular homeostasis during stressful conditions. In addition, Sestrins were shown to directly regulate autophagy through ULK1 and p62, as well as the subsequent control of the oxidative stress response through transcription factor Nrf2 [1]. Consequently, when Sestrins are expressed, cells cease growth and proliferation, conserving energy and nutrient resources for damage repair and survival.

In various cellular stress situations, Sestrins have been demonstrated to be essential for cell survival and the prevention of pathologies, as outlined in earlier research [1]. In particular, recent findings reveal that Sestrins are critical for adaptation to stress, damage repair, and the suppression of pathologies in muscle tissue. Additionally, Sestrins contribute significantly to exercise adaptation, which is a mild form of stress known to benefit overall organismal physiology. Consequently, Sestrins not only safeguard muscle tissue from stress-induced damage but also mediate the positive effects of low-level stressors, such as exercise.

Moreover, the crystal structure of Sestrin2 provides a physicochemical basis for understanding how Sestrin-family proteins exert their beneficial biochemical and physiological activities. Sestrins’ crystal structure reveals two distinct motifs that regulate separate pathways [8]. The first motif, a helix-turn-helix oxidoreductase motif, is crucial for Sestrins’ antioxidant activity, which directly scavenges oxygen radicals. The second motif, a helix-loop DD (Asp-Asp) motif, is vital for controlling various signaling pathways, such as GATOR1/2 interactions, mTORC1 suppression, and AMPK activation. These two motifs are located in separate domains of Sestrins, indicating that their functions are independent. In addition to these functional motifs, there is another motif that can bind to an amino acid leucine, suggesting that Sestrin function might be regulated through leucine binding [14]. Future research focused on elucidating the structure of Sestrins within multiprotein complexes could provide insights into the biochemical mechanisms underlying their functions. This knowledge could potentially pave the way for the development of novel therapeutics that mimic Sestrin activities, offering innovative treatment options for various muscle-related conditions and promoting overall muscle health.

These recent advancements present intriguing opportunities for leveraging the molecular advantages of Sestrins in upcoming therapeutic strategies. By capitalizing on their protective and adaptive functions, Sestrins could potentially be employed to support and preserve muscle health, especially in the context of exercise and physiological stress. Further exploration into the roles of Sestrins in muscle tissue will undoubtedly reveal novel insights and opportunities for the creation of groundbreaking treatments and interventions.

In this minireview, our primary focus is on the newly identified functions of Sestrins in skeletal and cardiac muscle tissues and how they contribute to the physiological homeostasis of these organs.

## 2. Regulation of Sestrin Expression in Skeletal Muscle

From initial studies on Sestrins in humans, it was realized that Sestrins are highly expressed in skeletal and cardiac muscle tissues [15], indicating their importance in maintaining the physiological balance of these organs. Sestrin expression was also abundant in the skeletal and cardiac muscle tissues of model organisms, such as mice [16], flies [17], and worms [18], suggesting the conserved role of Sestrins in these organs. The elevated Sestrin expression levels in muscle tissues are believed to result primarily from their strong expression in differentiated myofibers. Sestrin1, the isoform with the highest expression in muscle tissue, was found to be predominantly expressed in differentiated myotubes, while its expression in undifferentiated myoblasts remained minimal [16]. This suggests that the major contribution to high Sestrin expression in muscle tissues comes from myofiber expression, while the expression in other non-myocyte cells plays a relatively minor role.

Sestrin expression in skeletal muscle is primarily regulated by the levels of muscle activity. Too much activity (in the context of exercise) and too little activity (in the context of disuse) can modulate the levels of muscular Sestrin expression in different ways depending on the experimental settings. In addition, in mammals, different isoforms of Sestrins can be regulated differently in muscle. Sestrin1 is the predominant isoform expressed in differentiated skeletal muscle, but Sestrin2 and Sestrin3 are also highly expressed [15]. Notably, Sestrin expression is specifically high in mouse soleus muscle, which is enriched with oxidative fibers [16]. Both long-term and short-term treadmill exercise were found to increase the levels of Sestrin2 and Sestrin3 [19,20,21], while only acute exercise induced Sestrin1 and Sestrin2 [22] according to a different study. Sestrin3 expression in the quadriceps was strongly elevated by exercise training in high-fat-diet-fed obese mice [23]. In the mouse model of Sarcopenia, increased physical activity upregulated Sestrin2 expression, which was associated with improvements in muscle homeostasis [24]. Exercise also upregulated Sestrin levels in the liver [25] and gut [26], suggesting a systemic regulation mechanism. On the other hand, overload-induced skeletal muscle hypertrophy was associated with suppressed Sestrin2 levels [27]. In humans, Sestrin1 expression was upregulated in response to acute exercise [28]. Conversely, extensive disuse and inactivity reduced the level of Sestrin1 expression [29] and Sestrin3 expression [30] in mice and Sestrin2 expression [31] in humans. Human aging is also associated with the downregulation of Sestrins proteins [32]. Low circulating Sestrin2 levels predicted muscle force deficits [33] and Sarcopenia [34] in humans, suggesting Sestrin2 as a potential biomarker of frailty. However, higher circulating levels of Sestrin1 were associated with poorer physical function [35].

In general, Sestrin expression in skeletal muscle tissue is positively associated with physical activity levels; however, depending on the nature of the interventions and measuring schemes, there is sometimes an inverse correlation between the activity levels and Sestrin expression, indicating a complex mechanism regulating Sestrins in muscle.

## 3. Regulation of Sestrin Expression in the Heart

Not many studies on Sestrin expression in the heart with respect to exercise capacity exist. However, there are many studies examining Sestrin expression in the heart in the context of cardiac stress. For instance, Sestrin2 levels were found to be elevated in ischemic cardiomyocytes [36] and in cardiac macrophages during reperfusion-induced myocardial infarction [37]. However, myocardial ischemia/reperfusion injury was also reported to downregulate Sestrin1 expression in cardiomyocytes [38]. In response to pressure overload, Sestrin2 expression was significantly increased in mouse hearts during early phases, but this reverted back to normal levels at 8 weeks [39].

Doxorubicin is a well-known chemotherapy agent whose side effects include induction of cardiomyopathy. Doxorubicin was initially characterized to induce Sestrin2 expression in mice [40], but it was later shown to suppress Sestrin2 expression in rats [41]. Recently, it was shown that doxorubicin can induce Sestrin2 ubiquitination and degradation through MDM2 [42], independent of DNA damage-dependent transcriptional pathways that can induce Sestrin2. Therefore, the effect of doxorubicin can be variable depending on the context and timing of treatment.

Sestrin2 expression declines with age in mice, which is associated with increased susceptibility to myocardial injury [43]. Circulating Sestrin levels were elevated in human patients with hypertension [44], permanent atrial fibrillation [45], calcific aortic disease [46], and coronary heart diseases [47,48], suggesting the general association of Sestrins with cardiovascular diseases. Taking these results together, we can say that cardiovascular stress and pathologies are generally associated with increased Sestrin expression, while the development of chronic pathologies in certain contexts are associated with the downregulation of Sestrins, which further exacerbates the pathologies.

## 4. The Role of Sestrins in Exercise Adaptation and Benefits

**Figure 1:**
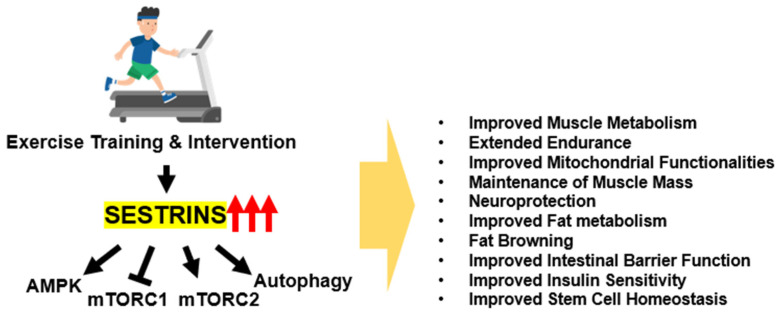
Role of Sestrins in the context of exercise. Sestrins mediate many metabolic benefits of exercise through modulation of multiple mechanisms, including AMPK-PGC, mTORC1-S6K, mTORC2-AKT, and autophagy pathways.

The exercise responsiveness of Sestrin expression suggests that it has a role in exercise physiology. Indeed, exercise produces many physiological changes in muscle and whole body metabolism, and considering the central role of Sestrin in metabolic homeostasis [49], it is not surprising that Sestrins play a major role in exercise physiology through multiple pathways (Figure 1).

The initial study on Sestrin’s function in exercise physiology was conducted using Drosophila and mouse models, and it revealed a conserved role. In both organisms, ablation of Sestrins strongly diminished exercise capacity, and in flies, overexpression of Sestrins in muscle strongly enhanced exercise capacity [16,50]. Mice deficient in Sestrin1 showed slightly decreased voluntary running capacity and reduced fat oxidation during forced exercise [16]. In addition, exercise improved glucose tolerance in WT mice but not in Sestrin1-deficient mice [16]. In contrast to the mild phenotype of Sestrin1 KO mice, mice deficient of all Sestrins (Sestrin1-3 TKO mice) exhibited much stronger phenotypes. Even though Sestrin1-3 TKO mice exhibited almost normal metabolism at baseline, they exhibited a strong deficit in oxidative respiration during exercise training, leading to an early loss of fat oxidation, physical exhaustion, and low VO_2_max [16]. Mechanistic studies performed using tissue sample analysis, Drosophila genetics, and in vitro myotube culture system suggested that Sestrin-regulated signaling pathways, such as AMPK-PGC1 and mTORC2-AKT pathways, are critical for Sestrins’ activities in upregulating metabolic capacity extending endurance [16]. In contrast, exercise deprivation in mice led to prominent muscle atrophy, which was exacerbated by Sestrin loss; Sestrin overexpression, however, protected against it [29]. Sestrin-mediated AKT upregulation is again shown to be critical for suppressing FoxO-dependent atrophic transcription and proteolytic muscle wasting. In addition, both endogenous and overexpressed Sestrin2 contributed to preventing myofiber type transition from slow-twitch to fast-twitch and preserved muscle mass in denervated atrophy [51]. Therefore, Sestrin is important for the functional capacity of skeletal muscle as well as the maintenance of muscle mass [52].

Subsequent studies in Sestrin2 and Sestrin3 showed that these isoforms are also critical for various benefits of exercise in multiple organ systems. Sestrin2 was previously shown to induce autophagy and attenuate insulin resistance in C2C12 myotubes [53] and to associate with muscular AMPK that is critical for the effect of exercise on insulin sensitivity [21], consistent with the exercise study described above [16]. Loss of Sestrin2 blunted the effect of exercise training in improving mitochondrial functionality in sarcopenic muscle, which, again, was dependent on AMPK signaling [54]. Sestrin2 was also important for mediating exercise effects in other organs. For instance, Sestrin2 loss attenuated the exercise-induced browning of white adipose tissue in mice [55,56]. This was thought to be mediated through increased production of Irisin/FNDC5, which could be induced by Sestrin2 through the AMPK-PGC1 axis. Sestrin2-deficient mice also did not get the benefits of exercise in protecting the epithelial barrier from obesogenic-diet-induced damages, again dependent on AMPK signaling [26]. However, exercise was still able to restore lipid homeostasis in obese Sestrin2-deficient mice [57], suggesting that other Sestrins may mediate the exercise benefits in that model. Indeed, Sestrin3-knockout mice were also shown to be defective in getting the metabolic benefits of exercise in high-fat-diet-fed obese mice, while Sestrin3 overexpression mimicked the exercise effects [23]. Most of the reported Sestrin activities depended on their control of AMPK-PGC and mTORC2-AKT pathways; therefore, even though Sestrins have many additional functions, these two pathways seem to play the most critical role in muscle fibers in the context of exercise adaptation and benefits.

Sestrins are important not only in differentiated myofibers but also in muscle stem cells. Sestrin expression in muscle stem cells was increased with voluntary wheel running exercise [24]. Loss of Sestrin1 and Sestrin2 resulted in defective stem cell homeostasis, leading to an accelerated depletion of muscle stem cells and subsequent impairment in muscle regeneration after injury [58]. In muscle stem cells, Sestrin-mediated regulation of mTORC1 turned out to play more critical roles, as Sestrins loss led to mTORC1 hyperactivation that disrupted muscle stem cell homeostasis and regenerative capacity [58].

Additional studies in flies furthered the understanding of the role of Sestrins in mediating the benefits of exercise beyond the skeletal muscle tissue. Spinocerebellar ataxias type (SCAs) 2, 3, and 6 are types of neurodegenerative diseases associated with polyglutamine (polyQ) expansion and expression. In Drosophila models of these SCAs, exercise was shown to suppress the neurodegenerative phenotypes and early death [59]. Interestingly, overexpressing Sestrin was sufficient to provide the benefits of exercise in suppressing degenerative phenotypes and early death exhibited by SCA2 model files, as did exercise [59]. Therefore, these results suggest that Sestrin is a tractable target for suppressing SCA pathologies, mimicking the effects of exercise.

Sestrins in skeletal muscle were also shown to be important for mediating the beneficial activities of adiponectin in improving insulin resistance. Although adiponectin has been shown to play a role in enhancing insulin sensitivity, the precise mechanisms behind this effect have yet to be fully understood. Sestrin2 was shown to play a critical role in adiponectin-induced AMPK phosphorylation and subsequent activation of insulin signaling and skeletal muscle insulin sensitization in mice [60].

Taken together, these findings suggest that Sestrin plays a critical role in exercise physiology. Sestrin ablation or deficiency impairs exercise capacity and the metabolic benefits of exercise, while Sestrin overexpression enhances exercise capacity and its benefits. Sestrin isoforms mediate exercise-induced benefits in multiple organ systems and are critical for insulin sensitivity, mitochondrial functionality, and lipid homeostasis. Therefore, Sestrins are essential for exercise adaptation and benefits.

## 5. The Role of Sestrins in Cardiac Stress Resistance and Protection

**Figure 2:**
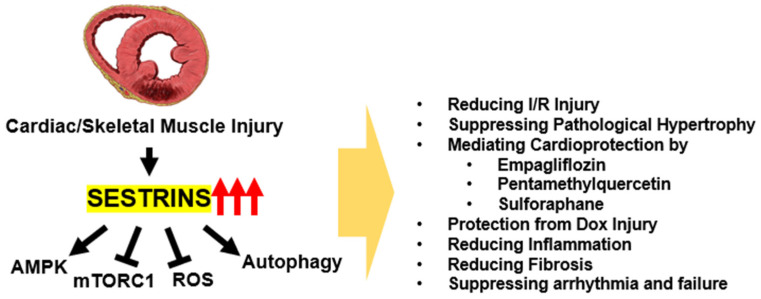
Protective Functions of Sestrins in Maintaining Cardiac and Skeletal Muscle Homeostasis. Sestrins exhibit a wide range of activities that combat stress-induced damage to maintain homeostasis in both cardiac and skeletal muscles. These actions collectively shield the muscles from various harmful insults, thereby promoting overall muscle health and function.

Sestrin expression is dynamically regulated by diverse types of stresses. Therefore, on the basis of its metabolism-regulating activities, it can be inferred that Sestrins play some role in maintaining homeostasis during stressful conditions. This is especially true in cardiac muscle, where the tissue is exposed to diverse types of injuries that lead to cardiac pathologies (Figure 2).

One of the most well-studied cardiac injury systems is the ischemia/reperfusion model, where the insult leads to the generation of myocardial infarction. Sestrins are upregulated during ischemia, and the induction of Sestrins is important for minimizing the ischemia/reperfusion-induced cardiac injury [36,43,61,62,63]. In the context of ischemia/reperfusion injury, Sestrins’ cardioprotection was mediated through their upregulation of the AMPK-PGC1 axis; Sestrins promoted the association between AMPK and its upstream kinase LKB1 [36,43,61,62,63]. Cardiac pressure overload can also induce pathological hypertrophy, which impairs the cardiac function. Overexpression of Sestrin1 was previously shown to protect against cardiac hypertrophy through induction of autophagy [64]. Interestingly, overexpression of Sestrin2 was also shown to exert a similar cardioprotective function in the pressure overload model [39]. Conversely, Sestrin2 deficiency aggravated the cardiac hypertrophy [65], confirming the protective role of endogenous Sestrins against pathological hypertrophy. Sestrins are also thought to mediate the cardioprotective activities of several therapeutic compounds, such as empagliflozin [66] and pentamethylquercetin [67,68]. Even without direct cardiac injury, loss of Sestrin2 by itself can provoke cardiac fibrosis and inflammatory damage during obesity, which is relatively mild in its Sestrin-proficient WT counterpart [69]. Likewise, in Drosophila, loss of Sestrin also provoked early onset development of cardiac arrhythmia and decreased cardiac output [17].

Sestrins also protect the heart from doxorubicin injury, which often arises as a side effect of chemotherapeutic treatments. In mice, concomitant loss of Sestrin1 and Sestrin2 strongly exacerbated cardiomyocellular damage induced by doxorubicin treatment, associated with dysregulation of the mTORC1-autophagy pathway [40]. In rats, Sestrin2 overexpression ameliorated the doxorubicin injury by promoting mitophagy, which is critical for eliminating dysfunctional mitochondria and improving mitochondrial function [41]. Sestrin2 was also critical for the cardioprotective activity of SIRT1, which was previously known to ameliorate doxorubicin injury [70,71]; Sestrin2 silencing abolished the protective effects of SIRT1 and resveratrol [42]. Interestingly, SIRT1 upregulated Sestrin2 by preventing its ubiquitination and degradation [42] in this model. In contrast to SIRT1, JMJD, a histone demethylase protein, aggravated doxorubicin injury in the heart through the downregulation of Sestrin2 expression [72]. Sestrins are also thought to protect against chronic chromium-induced cardiotoxicity, and they have been shown to mediate the protective role of sulforaphane in this context [73].

Sestrins were also shown to be important in the context of acute kidney injury (AKI)-associated cardiac damage and dysfunction. AKI can induce heart damage through oxidative stress, and Sestrins can suppress the damage. Experiments using cardiomyocyte-specific Sestrin2-knockout mice showed that metformin rescues AKI-induced cardiac injury through Sestrins and can improve a variety of indications, including systolic and diastolic function, fibrotic and cellular damage, and mitochondrial function. Importantly, metformin substantially upregulated Sestrin2 expression, suggesting that Sestrin2 mediates the cardioprotective effects of metformin [74].

These studies highlight the critical role of Sestrins in protecting the heart from a range of stress-induced injuries, including ischemia/reperfusion, pressure overload, and doxorubicin injury. Sestrins have been shown to regulate AMPK/mTORC1 signaling, autophagy, and mitophagy, which are all critical for maintaining cellular homeostasis and preventing cellular damage. Additionally, Sestrins can also mediate the cardioprotective effects of other therapeutic compounds and signaling pathways. The regulation of Sestrins by SIRT1 and JMJD further emphasizes the intricate interplay among these pathways in maintaining cardiac health.

## 6. Conclusions and Perspectives

Sestrin, a protein originally characterized for its high expression in skeletal and cardiac muscle tissues, has recently become the subject of physiological research regarding its involvement in muscle physiology. Early indications seem promising for Sestrin as a potential target for pharmaceuticals aimed at providing the physiological benefits of exercise and protecting against the detrimental effects of cardiac stress. Research has already demonstrated that Sestrin is a crucial mediator of exercise and other therapeutic agents that can enhance both health and lifespan.

There are several avenues for future studies to explore the beneficial activities of Sestrins. First, the molecular mechanisms behind Sestrin’s effects must be further studied. Despite the solving of Sestrin’s structure, how Sestrin affects its downstream targets remains unclear, hindering our understanding of its biochemical actions. The physiological oxidoreductase target of Sestrin is unknown and should be a key aspect of its antioxidant activity. Additionally, the mechanism by which Sestrin activates AMPK is also unclear, even though AMPK appears to be the most important target in controlling muscular and cardiovascular physiology. Research has shown that the GATOR1/2 complexes are crucial for mediating Sestrin’s mTORC1-suppressing and mTORC2-activating activities, but how Sestrin modulates these complexes is still unknown. It is also unknown how Sestrin interacts structurally with other partners involved in regulating autophagy and mitophagy. These biochemical details must be characterized to fully understand the basis of Sestrin’s function.

Second, the regulation of Sestrin’s activities should be further investigated. Given the delicate function of Sestrins, it is likely that, in addition to transcriptional regulation, there are additional post-transcriptional mechanisms at play. It has been suggested that Sestrins are regulated by leucine ligand binding, phosphorylation, and ubiquitination, but how these regulations contribute to muscle physiology has not yet been studied through genetic methodologies. Regulation of AMPK and mitochondrial energetics, integrity, and homeostasis seems to be crucial for Sestrin’s physiological function. Therefore, how Sestrin’s post-transcriptional regulation contributes to its beneficial activities in skeletal and cardiac muscle must be more thoroughly understood. These mechanistic details can lead to the development of novel therapeutics that mimic the effects of exercise or protect the cardiovascular system from physical or chemical damage. It is also possible that Sestrins may play a crucial role in some of the interventions already used to improve skeletal and cardiac health, as demonstrated by the case of exercise.

Finally, there is a growing need to investigate the function of Sestrin in various contexts of muscle disease. One such area of interest is Duchenne muscular dystrophy (Dmd), a progressive muscle degeneration disorder that currently has no cure. Expanding our understanding of Sestrins’ potential role in treating Dmd may pave the way for breakthroughs in therapeutic strategies. For instance, previous studies using mouse models have demonstrated that upregulating either AMPK [75,76] or AKT [77] signaling could be beneficial for ameliorating Dmd pathologies. Since Sestrins have activities in activating AMPK and AKT and can produce effects mimicking the metabolic benefits of exercise without actual exercise that can produce physical damages to myofibers, they could have potential activities towards treating Dmd. Likewise, Sestrin’s beneficial activities in improving muscle function and metabolism might be utilized to treat incurable muscle diseases, opening new directions for future research into novel therapeutics.

In conclusion, the growing body of research on Sestrin highlights its potential as a critical player in muscle physiology and as a promising therapeutic target for improving health and lifespan. As we continue to unravel the molecular mechanisms, regulatory processes, and functional implications of Sestrin in various muscle disease contexts, our understanding of its biochemical actions will deepen, enabling the development of novel pharmaceuticals that harness Sestrin’s unique properties. These therapeutics may provide valuable benefits for a wide range of individuals, including those suffering from muscle degenerative diseases or cardiovascular issues or those seeking the physiological advantages of exercise without the physical demands. By expanding our knowledge of Sestrin, we can ultimately unlock new possibilities for maintaining and enhancing muscle health, improving quality of life, and extending longevity.

## Data Availability

Not applicable.
